# Fractional flow reserve use in coronary artery revascularization: A systematic review and meta-analysis

**DOI:** 10.1016/j.isci.2023.107245

**Published:** 2023-07-03

**Authors:** Jorge Sanz Sánchez, Julio I. Farjat Pasos, Julia Martinez Solé, Bilal Hussain, Sant Kumar, Mohil Garg, Mauro Chiarito, Andrea Teira Calderón, Jose A. Sorolla-Romero, Mauro Echavarria Pinto, Eun-Seok Shin, José Luis Diez Gil, Ron Waksman, Tim P. van de Hoef, Hector M. Garcia-Garcia

**Affiliations:** 1 Hospital Universitari i Politecnic La Fe, Valencia, Spain; 2 Centro de Investigación Biomedica en Red (CIBERCV), Madrid, Spain; 3 Institut universitaire de cardiologie et de pneumologie de Québec, Quebec, Canada; 4 Internal Medicine, The Brooklyn Hospital Center, Brooklyn, NY, USA; 5 MedStar Washington Hospital Center, Washington, DC, USA; 6 IRCCS Humanitas Research Hospital, Rozzano, Milan, Italy; 7 Hospital Universitario Marqués de Valdecilla, Santander, Spain; 8 Hospital General ISSSTE Querétaro, Querétaro, México; 9 Universidad Autónoma de Querétaro, Querétaro, México; 10 Division of Cardiology, Department of Internal Medicine, Ulsan University Hospital, University of Ulsan College of Medicine, Ulsan, Korea; 11 Department of Cardiology, University Medical Center, Utrecht, the Netherlands

**Keywords:** Cardiovascular medicine, Pathology, Surgery

## Abstract

Fractional flow reserve (FFR)-guided percutaneous coronary intervention (PCI) is recommended in revascularization guidelines for intermediate lesions. However, recent studies comparing FFR-guided PCI with non-physiology-guided revascularization have reported conflicting results. PubMed and Embase were searched for studies comparing FFR-guided PCI with non-physiology-guided revascularization strategies (angiography-guided, intracoronary imaging-guided, coronary artery bypass grafting). Data were pooled by meta-analysis using random-effects model. 26 studies enrolling 78,897 patients were included. FFR-guided PCI as compared to non-physiology-guided coronary revascularization had lower risk of all-cause mortality (odds ratio [OR] 0.79 95% confidence interval [CI] 0.64–0.99, I^2^ = 53%) and myocardial infarction (MI) (OR 0.74 95% CI 0.59–0.93, I^2^ = 44.7%). However, no differences between groups were found in terms of major adverse cardiac events (MACEs) (OR 0.86 95% CI 0.72–1.03, I^2^ = 72.3%) and repeat revascularization (OR 1 95% CI 0.82–1.20, I^2^ = 43.2%). Among patients with coronary artery disease (CAD), FFR-guided PCI as compared to non-physiology-guided revascularization was associated with a lower risk of all-cause mortality and MI.

## Highlights

• FFR-guided was associated with a lower risk of all-cause mortality and MI
• No differences were observed in terms of MACE and repeat revascularization
• Large-scale clinical trials are required to confirm these results

## Graphical abstract

**Figure:**
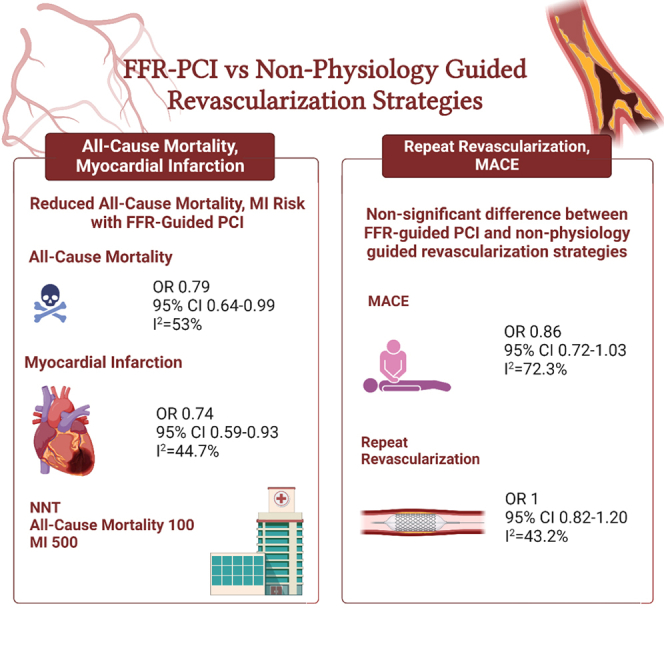

## Introduction

Fractional flow reserve (FFR) was developed in the 1990s to determine the functional significance of angiographically apparent coronary artery stenosis using intracoronary pressure measurements.^1^ Nowadays, FFR has become the standard to guide decisions on percutaneous coronary revascularization, achieving class 1A recommendation in both US^2^ and European^3^ guidelines. Alongside the growing evidence supporting FFR-guided revascularization strategies,^4^^,^^5^^,^^6^ several other revascularization approaches, such as angiography,^7^^,^^8^ intravascular ultrasound,^9^^,^^10^ and optical coherence tomography-guided^11^^,^^12^^,^^13^ revascularization, have been proposed to optimize revascularization strategies in terms of functional/imaging results, but also in terms of short- and long-term clinical outcomes. Even though these different strategies have been extensively investigated, comparative studies against the current clinical standard, FFR, are scarce.^14^ This becomes of increasing importance in light of recent evidence-based practice trials using FFR-guided percutaneous coronary intervention (PCI) with new-generation drug-eluting stents, where no benefit of physiological guidance could be documented.^15^^,^^16^ Overall, our knowledge on the impact of an FFR-guided PCI strategy on clinical outcomes compared to angiography or invasive imaging-guided revascularization strategies in contemporary practice has resulted in conflicting results. Hence, we conducted a systematic review and meta-analysis of randomized and observational trials from studies of FFR-guided PCI vs. other revascularization strategies (angiography-guided PCI, intracoronary imaging-guided PCI, coronary artery bypass grafting [CABG]), aimed at comparing FFR-guided PCI in terms of its benefits on clinical outcomes against different revascularization approaches.

## Results

**Figure 1:**
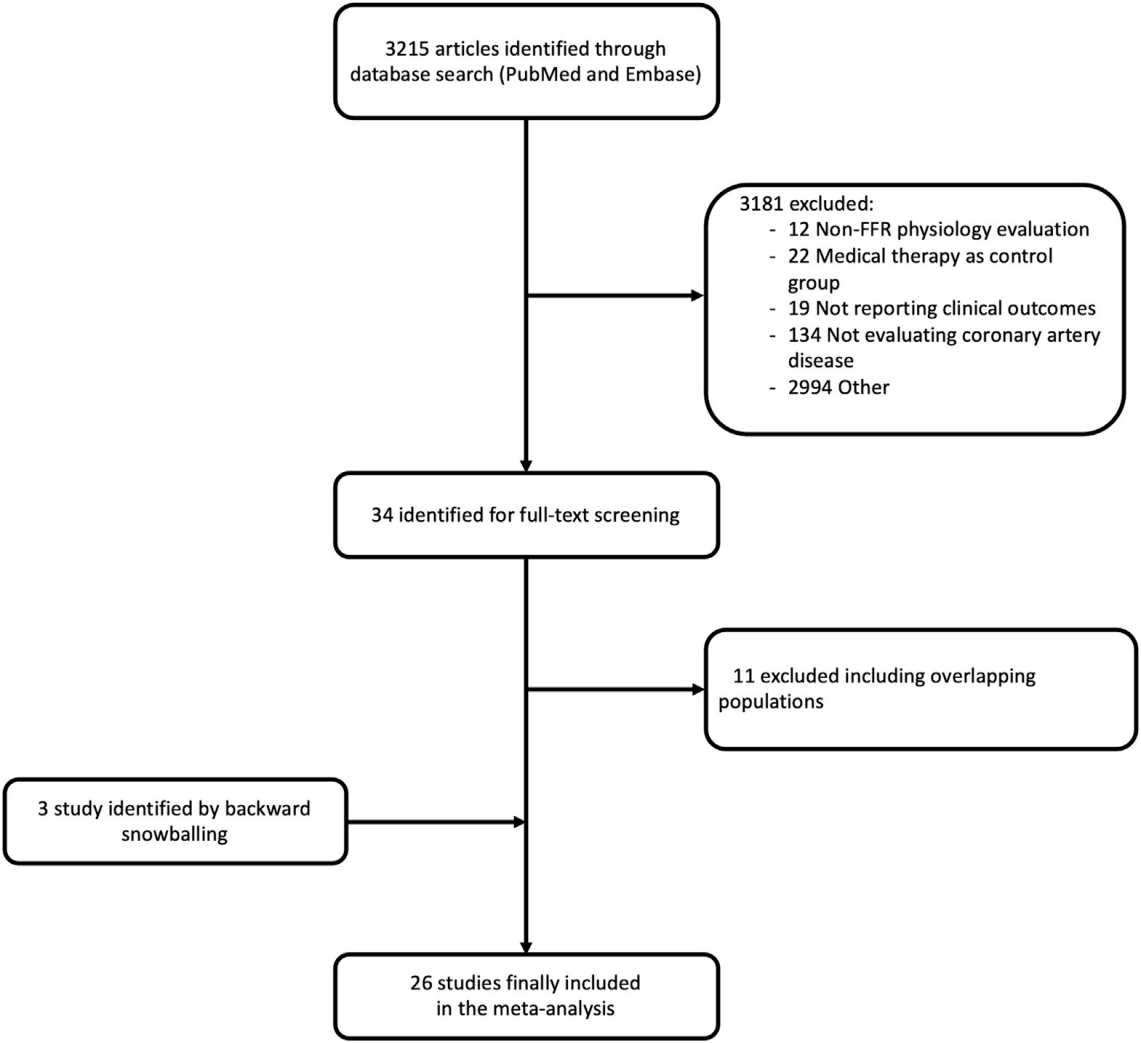
Flow chart of the study selection process FFR, fractional flow reserve.

Figure 1 displays the PRISMA study search and selection process. A total of 26 studies, 12 RCTs and 14 observational, were identified and included in this study. The main features of included trials are presented in Table 1.

**Table 1:** Key study features

Study	Study design	Year of publication	N of patients			Control	FFR threshold	Multicentre	Follow-up
			Overall	FFR-guided	Non-physiology				
De la Torre et al.^51^	Observational	2013	800	400	400	Intracoronary imaging-guided	0.75	Yes	24 months
Defer DES^52^	RCT	2015	229	114	115	Angiography-guided	0.75	Yes	60 months
Di Gioia et al. 2020^37^	Observational	2020	418	209	209	Coronary artery bypass grafting	0.8	Yes	60 months
DK-CRUSH VI^53^	RCT	2015	320	160	160	Angiography-guided	0.8	Yes	12 months
Elkady et al.^54^	RCT	2021	90	30	60	Angiography-guided	0.8	No	6 months
FAME^31^	RCT	2015	1,005	509	496	Angiography-guided	0.8	Yes	60 months
FAME 3^29^	RCT	2021	1,500	757	743	Coronary artery bypass grafting	0.8	Yes	12 months
FAMOUS NSTEMI^20^	RCT	2014	350	176	174	Angiography-guided	0.8	Yes	12 months
FLAVOR^21^	RCT	2022	1,682	838	844	Intracoronary imaging-guided	0.8	Yes	24 months
FLOWER MI^15^	RCT	2021	1,171	590	581	Angiography-guided	0.8	Yes	12 months
FORZA^35^	RCT	2020	350	176	174	Intracoronary imaging-guided	0.8	No	13 months
Fröhlich et al.^22^	Observational	2014	41,688	2,767	37090 1831	Angiography-guided Intracoronary imaging-guided	0.8	Yes	84 months
FUTURE^16^	RCT	2021	927	460	467	Angiography-guided	0.8	Yes	12 months
Di Giogia et al. 2016^55^	Observational	2015	318	106	212	Angiography-guided	0.8	No	60 months
Hu et al.^23^	Observational	2015	732	366	366	Angiography-guided	0.8	No	13 months
Koo et al.^56^	Observational	2008	220	110	110	Angiography-guided	0.75	No	12 months
Li et al.^24^	Observational	2013	7,358	1,090	6,238	Angiography-guided	0.75	No	84 months
Lunardi et al.^25^	RCT	2019	216	94	122	Angiography-guided	0.8	No	24 months
Nam et al.^57^	RCT	2010	167	83	94	Intracoronary imaging-guided	0.8	No	12 months
Parikh et al.^26^	Observational	2019	17,989	2,967	15,022	Angiography-guided	0.8	Yes	12 months
Puymirat et al.^27^	Observational	2012	717	222	495	Angiography-guided	0.8	No	60 months
Quintella et al.^58^	RCT	2018	70	34	35	Angiography-guided	0.75	No	12 months
Serafino et al.^59^	Observational	2013	223	65	158	Angiography-guided	0.8	No	48 months
Wongpraparu et al.^60^	RCT	2005	137	57	80	Angiography-guided	0.75	No	30 months
Zhang et al.^61^	RCT	2018	220	110	110	Angiography-guided	0.8	No	12 months

FFR, fractional flow reserve; RCT, randomized clinical trial.

### Baseline characteristics

Baseline characteristics of patients included are summarized in Table 2. A total of 78,897 patients were enrolled, of whom 12,490 (15.8%) underwent FFR-guided PCI, 62,091 (78.7%) angiography-guided PCI, 3,364 (4.2%) intracoronary imaging-guided PCI, and 952 (1.2%) CABG.

**Table 2:** Baseline clinical characteristics of included patients

Study	Age (years)	Male (%)	Diabetes (%)	Hypertension (%)	Dyslipidemia (%)	Prior CVA (%)	Prior MI (%)	ACS (%)	PAD (%)	Median number of affected vessels
De la Torre et al.^51^	65,6	74,4	38,6	71,4	56	–	19,8	63,4	–	–
Defer DES^52^	62	73	26	64	70	–	19	51	–	1,8
Di Gioia et al. 2020^37^	67,3	75,5	100	70,5	73	–	10	10,5	20	2,6
DK-CRUSH VI^53^	65,3	74,1	28,5	70,4	18,5	12,5	9,7	78,4	1,9	2,1
Elkady et al.^54^	61,2	82	43,3	61,1	–	–	–	100	–	2
FAME^31^	64,2	75	23,5	63	71,7	–	35,2	32,5	–	–
FAME3^29^	65.2	82,4	28,5	73,1	70,3	7	33,4	39,2	–	–
FAMOUS NSTEMI^20^	62	74,3	14,9	45,5	36,3	6,9	13,2	100	8	1,8
FLAVOR^21^	65.1	70.6	32.9	68.2	78.6	–	5.6	29.5	–	–
FLOWER MI^15^	62,2	83,1	16,3	44,3	40,4	2,9	6,7	100	3,4	2,2
FORZA^35^	68,5	74,6	35,5	85,5	76,5	–	24,4	19,4	–	–
Fröhlich et al.^22^	65,6	74,2	23,5	56,8	53,6	2,1	34,4	40,5	3	1,4
FUTURE^16^	65,5	83,5	31,5	59,5	60,5	4,5	20,5	46,5	–	2,5
Di Giogia et al. 2016^55^	73	70	24	57	54,7	–	–	8	11	1,82
Hu et al.^23^	63,5	77	28,6	68,7	30,4	–	15,9	57,6	–	1,86
Koo et al.^56^	62,5	69	28,5	–	–	–	–	52,5	–	–
Li et al.^24^	67,5	69,2	29,5	78,25	77,9	10,6	30,1	11,8	11,2	1,1
Lunardi et al.^25^	84	47,7	31,5	91,7	–	6	19,5	–	–	1,5
Nam et al.^57^	62,5	62,4	23,6	46,7	15,3	–	–	59	–	–
Parikh et al.^26^	65.8	97.2	44.6	89.3	85.9	15	22.1	0	16.2	–
Puymirat et al.^27^	71,7	64,9	30,8	63,1	65,5	8,4	–	17,6	10	1,1
Quintella et al.^58^	62	68,1	35,8	73,9	72,5	–	21,7	39,1	–	2,2
Serafino et al.^59^	70,4	77	26,7	57	62,5	11,1	35	23,7	19,4	–
Wongpraparu et al.^60^	60,3	55,3	37,7	73,3	62,5	–	–	–	–	2,3
Zhang et al.^61^	70	70	34,6	74,6	83,2	–	21,4	100	–	–

Data are represented as mean.

ACS, acute coronary syndrome; CVA, cerebrovascular disease; MI, myocardial infarction; PAD, peripheral artery disease.

### Clinical outcomes

**Figure 2:**
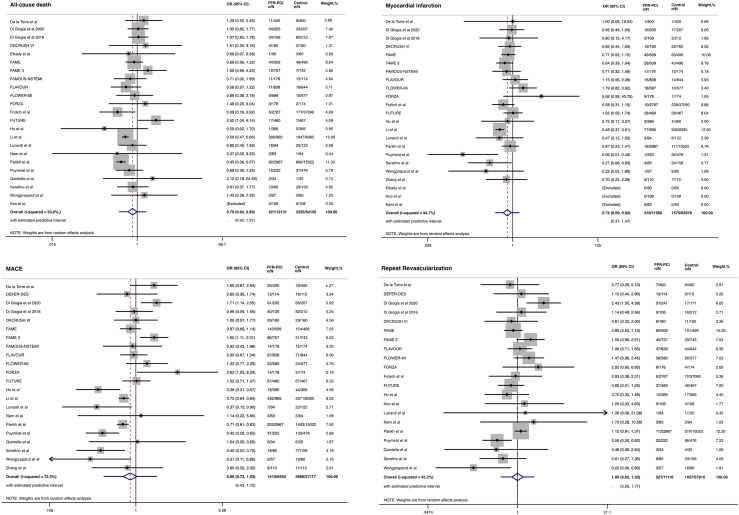
Clinical outcomes in patients undergoing FFR-guided PCI as compared to non-physiology-guided revascularization strategies CI, confidence interval; FFR, fractional flow reserve; OR, odds ratio; PCI, percutaneous coronary intervention.

Patients undergoing FFR-guided PCI as compared to those undergoing non-physiology-guided coronary revascularization had a lower risk of all-cause mortality (OR 0.79 95% CI 0.64–0.99, I^2^ = 53%) and MI (OR 0.74 95% CI 0.59–0.93, I^2^ = 44.7%). However, no differences between groups were found in terms of MACE (OR 0.86 95% CI 0.72–1.03, I^2^ = 72.3%) and repeat revascularization (OR 1 95% CI 0.82–1.20, I^2^ = 43.2%). **(**Figure 2**)**. NNT to prevent one death was 100 patients, and NNT to prevent one MI was 500 patients (Graphical abstract). Nevertheless, high heterogeneity was found, resulting in prediction intervals that showed a possible null effect on the risk of all-cause mortality and MI **(**Figure 2**)**.

### Risk of bias assessment

Tables S2 and S3 summarize the results of the risk of bias assessment. Among observational studies, 4 presented moderate overall risk of bias and 10 were considered at serious overall risk of bias. Among RCTs, 1 was considered at low overall risk of bias and 11 presented some concerns.

### Subgroup analysis

A stratified analysis according to the revascularization strategy revealed that the reduced risk of all-cause mortality among patients undergoing FFR-guided PCI was mainly driven by a significant lower risk of death in studies with angiography-guided PCI as comparative arm (OR 0.73 95% CI 0.58–0.92, I^2^ = 53.3%), despite lack of significant interaction for the comparative arm (p_interaction_ = 0.14). Similarly, the reduced risk of MI in patients undergoing FFR-guided PCI was only shown in studies with angiography-guided PCI as comparative arm (OR 0.69 95% CI 0.53–0.90, I^2^ = 49%, p_interaction_ = 0.56). In addition, patients undergoing FFR-guided PCI as compared to patients undergoing angiography-guided PCI had a statistically significant lower risk of MACE (OR 0.73 95% CI 0.62–0.85, I^2^ = 53.6%, p_interaction_ = 0.001). Of note, high heterogeneity and wide prediction intervals suggested a possible null effect on the risk of all-cause mortality, MI, and MACE. Conversely, patients undergoing FFR-guided PCI as compared with patients undergoing CABG showed a statistically significant higher risk of MACE (OR 1.65 95% CI 1.26–2.16, I^2^ = 0%, p_interaction_ = 0.001) and repeat revascularization (OR 1.88 95% CI 1.22–2.90, I^2^ = 25%, p_interaction_ = 0.004) (Figure S5**)**.

After stratifying according to study design, the reduced risk of all-cause mortality (OR 0.69 95% CI 0.55–0.87, I^2^ = 47.8%, p_interaction_ = 0.12), MI (OR 0.60 95% CI 0.45–0.80, I^2^ = 32.3%, p_interaction_ = 0.05), and MACE (OR 0.72 95% CI 0.57–0.92, I^2^ = 76.1%, p_interaction_ = 0.05) in patients undergoing FFR-guided PCI as compared to other revascularization strategies was driven by observational studies. High heterogeneity was found in prediction intervals resulting in a possible null effect on the risk of all outcomes (Figure S6**).**

### Sensitivity analyses

At leave-one-out sensitivity analysis, when removing any of the following studies FAMOUS-NSTEMI,^20^ FLAVOR,^21^ Frolich et al.,^22^ Hu et al.,^23^ Li et al.,^24^ Lunardi et al.,^25^ Parikh et al.,^26^ or Puymirat et al.^27^ the risk of all-cause mortality was no longer significantly reduced in patients undergoing FFR-guided PCI. Also when removing the Di Giogia et al. 2020^28^ or the FAME (fractional flow reserve versus angiography for guiding percutaneous coronary intervention) 3,^29^ a lower risk of MACE emerged in patients undergoing FFR-guided PCI. Results were consistent with the primary analysis for the rest of outcomes by iteratively removing one study at a time (Table S5. Leave-one sensitivity analysis for all-cause death, related to STAR Methods (Quantification and Statistical Analysis), Table S6. Leave-one sensitivity analysis for myocardial infarction, related to STAR Methods (Quantification and Statistical Analysis), Table S7. Leave-one sensitivity analysis for repeat revascularization, related to STAR Methods (Quantification and Statistical Analysis), Table S8. Leave-one sensitivity analysis for MACE, related to STAR Methods (Quantification and Statistical Analysis)**)**. Meta-regression showed impact of number of diseased vessels and follow-up duration on all-cause death and MI. No impact of percentage of population presenting with ACS, number of diseased vessels, time of follow-up, and treatment effect was shown for the remaining outcomes (Table S4**).** Publication bias was detected for all-cause mortality as shown by Harbord test (p = 0.005). Funnel-plot distributions and Harbord test of the remaining outcomes indicated absence of publication bias and small study effect (Figures S1–S4).

## Discussion

In this study we evaluated FFR-guided PCI with non-physiology-guided revascularization strategies (angiography-guided PCI, intracoronary imaging-guided PCI, CABG) in patients with CAD. The main findings of this study can be summarized as follows.

(1) The risk of all-cause mortality and MI in patients undergoing FFR-guided PCI is lower as compared with those undergoing non-physiology-guided revascularization, with an NNT of 100 patients to prevent one death. However, high heterogeneity was found, resulting in prediction intervals that showed a possible null effect on the risk of death and MI.
(2) The risk of repeat revascularization and MACE does not differ between patients treated with FFR-guided PCI and those treated with non-physiology-guided revascularization strategies.

### FFR evidence leading to current guidelines recommendations

There is emerging evidence supporting the role of FFR to assess lesion significance and guide revascularization decisions. This is endorsed by the fact that the class of recommendation for FFR to assess intermediate (50%–70%) coronary artery stenosis and direct revascularization plan has been upgraded from a 2A recommendation^30^ to a 1A recommendation in 2021 US guidelines^2^ and 2018 European guidelines.^3^ Several large-scale trials have established the efficacy and superiority of FFR-guided PCI as compared to angiography-guided PCI in terms of mortality, MI, or repeat revascularization. The DEFER (fractional flow reserve to determine the appropriateness of angioplasty in moderate coronary stenosis) trial has shown that deferral of PCI of a functionally nonsignificant stenosis is not associated with increased risk of MACE at long-term follow-up.^4^ The FAME 1 trial compared FFR with angiography to guide revascularization and found that FFR-guided PCI was associated with reduced adverse cardiac events at 1-year follow-up,^5^ while at 5-year follow-up long-term outcomes of FFR-guided PCI led to equivalent results in terms of MACE compared with angiography-guided PCI, but with the use of fewer stents.^31^ The FAME 2 trial determined that FFR-guided PCI plus medical therapy was associated with lower rates of death, MI, or urgent revascularization as compared to medical therapy alone for patients with functionally significant stenoses,^6^ although still half of patients with abnormal FFR results did not experience adverse events or required revascularization during 5 years of follow-up.^32^ Other trials have questioned the effectiveness of FFR-guided PCI in different clinical settings and found conflicting results. Many studies and meta-analyses have compared FFR-guided PCI with angiography-guided PCI;^33^^,^^34^ however, in recent years an interest has emerged in comparing FFR-guided PCI with imaging-guided PCI and CABG and some important trials have been conducted.^21^^,^^29^^,^^35^ Therefore, it is important to compare the role of FFR-guided PCI with all available evidence on different revascularization strategies to provide a comprehensive and quantitative assessment of evidence about the contemporary efficacy of FFR-guided PCI which we aimed to achieve with this meta-analysis.

### Ongoing FFR controversy

The FAME 3^29^ trial documented that FFR-guided PCI was associated with higher risk of MACE as compared to CABG in patient with multivessel CAD, hence establishing that FFR-guided PCI was not noninferior to CABG.^29^ The FUTURE (FUnctional Testing Underlying coronary REvascularization) trial compared an FFR-guided revascularization strategy to a traditional angiography-guided strategy without FFR in all-comer multivessel CAD patients.^16^ The trial was prematurely stopped due to an observed significantly higher all-cause mortality in the FFR-guided group. However, this observation was not confirmed by the intention-to-treat analysis at 1-year follow-up. At 1-year follow-up, no significant difference between FFR-guided based strategy and angiographic-guided strategy was found in terms of MACE.^16^ The FLOWER-MI (flow evaluation to guide revascularization in multivessel ST-elevation myo- cardial infarction) trial also deduced that in patients with ST-segment elevation MI undergoing complete revascularization, an FFR-guided strategy did not show any benefit over an angiography-guided strategy with respect to the risk of death, MI, or urgent revascularization at 1 year.^15^ Furthermore, studies have suggested that FFR-guided PCI results in lower stroke events than non-physiology-guided PCI.^36^ However, Gioia et al., 2020, only observed this decreased stroke risk at 1 year for FFR-guided PCI as compared to angiography-guided strategy, but no difference was seen at 5-year follow-up.^37^ Hence, studies conducted so far have given mixed results with some of them establishing FFR as gold standard and others questioning its effectiveness and positive outcomes. Consequently, there is an urgent need to pool and analyze the results from these trials to institute the efficacy of FFR-guided PCI which we aimed to accomplish with this meta-analysis.

### Comparison with current results

In the present investigation including 26 studies and 78,897 patients, patients undergoing FFR-guided PCI as compared to other revascularization strategies (angiography-guided PCI, intracoronary imaging-guided PCI, or CABG) were associated with a reduced risk of all-cause mortality, which is mechanistically explained by a significant reduction in the risk of MI. Like the FAME 1 trial^5^ and unlike FUTURE^16^ and FLOWER-MI trials,^15^ our subgroup analysis showed that FFR-guided PCI was associated with statistically significant lower risk of MI and MACE as compared to angiography-guided PCI. In addition, FFR-guided PCI has been associated with the implantation of fewer stents, as compared to an angiography-guided PCI.^3^ Concordant with FAME 3 trial,^29^ our subgroup analysis concluded that FFR is associated with higher risk of MACE and repeat revascularization as compared to CABG.

Despite our analysis proving superiority of FFR-guided PCI compared to non-physiology-guided revascularization strategies altogether in hard endpoints such as all-cause mortality and MI, some aspects are worth highlighting. The reduced risk of all-cause mortality was not consistent neither at leave-one-out sensitivity analysis nor at subgroup analysis. In addition, wide prediction intervals showed possible null effect on the risk of all-cause mortality. Similarly, important limitations were shown in the lower risk of MI in patients undergoing FFR-PCI such as wide prediction intervals, and subgroup analysis results were non-consistent with this risk reduction. In addition, different definitions of MI have been used across studies influenced by the year the trial was conducted. As a result, the diagnosis of MI and its impact on mortality after FFR-guided PCI or other revascularization strategies are highly variable according to the MI definition used in the trial. This has been highlighted by Maron et al.^38^ who compared the invasive and conservative strategies for the management of patients with stable CAD showing that the risk of death was sensitive to the definition of MI used.^38^ Furthermore, the appropriateness of non-fatal MI as a surrogate measure to predict mortality has been recently questioned. O’Fee et al.^39^ have recently conducted a meta-analysis and concluded that non-fatal MI did not meet the threshold to be designated as a surrogate measure for all-cause or cardiovascular mortality in primary, secondary, and mixed prevention and revascularization trials.^39^^,^^40^ Despite the important limitations of the aforementioned study such as including trials that used time-to-event statistics for composite endpoints which considers only the first event,^41^ the risk reduction in MI instituted by our study should be interpreted with caution due to the reasons discussed above.

### FFR use in ACS

It should be highlighted that the class 1A recommendation for FFR to evaluate coronary artery stenosis and direct revascularization strategies applies to patients presenting with stable CAD.^2^^,^^3^ In an acute setting, hemodynamics and FFR evaluation differs from that of patients with stable CAD. Despite some studies showing that the addition of FFR-guided revascularization of non-infarct-related arteries is associated with lower risk of all-cause mortality and MACE as compared to patients with ST-segment elevation MI who are only treated with PCI for the infarct-related artery,^42^^,^^43^ other studies have questioned its usefulness in patients presenting with ACSs.^44^^,^^45^ Different trials and meta-analyses have shown that deferral of revascularization based on non-ischemic FFR in patients presenting with ACSs is associated with higher risk of mortality and MACE as compared to stable CAD patients, which has been attributed to higher rates of unplanned revascularization.^44^^,^^45^^,^^46^^,^^47^ A proposed mechanism is that in an acute setting, MI causes microvascular dysfunction not only limited to the myocardium supplied by the culprit artery, reducing the hyperemic response to a vasodilating agent like adenosine, and FFR, which is a hyperemic index, tends to underestimate the true severity of stenosis leading to false-negative FFR.^48^ Hitherto, FFR threshold to define a significant stenosis in ACS patients’ needs to be investigated and FFR values derived from stable CAD should be used with caution for decision making in ACS patients.

### FFR versus intracoronary imaging for PCI guidance

Finally, intracoronary imaging with intravascular ultrasound or optical coherence tomography has the potential to identify vulnerable plaques at risk of future events and physiologically nonsignificant lesions requiring preventive treatment and even guide revascularization deferrals for patients with ACSs.^49^ The FORZA (fractional flow reserve vs. optical coherence tomography to guide revas- cularization of intermediate coronary stenoses) trial randomized patients to undergo FFR-guided PCI or optical coherence tomography-guided PCI.^35^ At 13-month follow-up optical coherence tomography-guided PCI was associated with lower risk of MACE and angina as compared to FFR-guided PCI.^35^ COMBINE OCT-FFR (combined optical coherence tomography morphologic and fractional flow reserve hemodynamic assessment of non- culprit lesions to better predict adverse event outcomes in diabetes mellitus patients) trial divided the diabetic patients with FFR-negative lesions into two groups based on the presence or absence of ≥1 thin-cap fibroatheroma lesion assessed by optical coherence tomography.^50^ This study deduced that presence of thin-cap fibroatheroma was associated with five times higher risk of MACE as compared to its absence despite the lack of ischemia,^50^ thus, establishing the significance of studying the anatomical plaque characteristics. These findings highlight the increased frequency of vulnerable plaques in patients with multivessel disease and hint at the future need to use intracoronary imaging to guide the revascularization of non-culprit lesions in ACS patients with multivessel disease.

As shown by the present investigation, the one-size-fits-all approach should not be applied when evaluating the role of FFR to guide revascularization as patients with multivessel disease and those presenting with ACS represent different clinical scenarios where the impact of FFR-guided PCI is yet under debate.

### Limitations

The results of our investigation should be interpreted in light of some limitations. First, this is a study-level meta-analysis providing average treatment effects; the lack of patient-level data from the included studies prevents us from assessing the impact of baseline clinical and procedural characteristics on treatment effects. Second, minor differences in definition were present for some endpoints, limiting the reliability of effect estimates. Finally, the limited number of studies in some subgroups (i.e., those undergoing intracoronary imaging-guided PCI) may reduce the power for detecting significant differences between groups.

### Research in context panel

#### Evidence before this study

Recently published studies comparing FFR-guided PCI with non-physiology-guided revascularization strategies (angiography-guided, intracoronary imaging-guided, CABG) have reported conflicting result. Some trials have established FFR as gold standard for coronary revascularization (FAME, FAME 2) while others have questioned its effectiveness and positive outcomes (FAME 3, FUTURE, FLOWER MI). Consequently, there is an urgent need to pool and analyze the results from published evidence to institute the efficacy of FFR-guided PCI. Therefore, we performed a systematic review and meta-analysis of studies comparing FFR-guided PCI with non-physiology-guided revascularization strategies.

#### Added value of this study

This is the most comprehensive systematic review and meta-analysis of evidence evaluating FFR-guided PCI versus non-physiology-guided revascularization strategies (angiography-guided, intracoronary imaging-guided, CABG). We included findings of 78,897 patients from 26 studies. Patients undergoing FFR-PCI as compared to those undergoing non-physiology-guided revascularization strategies were associated with a lower risk of all-cause mortality and MI, while no differences between groups were found in terms of MACE and repeat revascularization. The NNT to prevent one death was 100 patients.

#### Implications of all the available evidence

In patients with CAD undergoing coronary revascularization, FFR-guided PCI was associated with a lower risk of all-cause mortality, which is mechanistically explained by a reduced risk of MI. Notwithstanding, the results should be interpreted with caution. The one-size-fits-all approach should not be applied when evaluating the role of FFR to guide revascularization as patients with multivessel disease and those presenting with ACS represent different clinical scenarios where the impact of FFR-guided PCI is yet under debate.

### Conclusions

Among patients with CAD, FFR-guided PCI as compared to non-physiology-guided revascularization strategies was associated with a lower risk of all-cause mortality and MI. No differences between groups were shown in the risk of MACE and repeat revascularization. However, these findings should be interpreted with caution given high heterogeneity leading to prediction intervals that show a possible null effect on the risk of all-cause death and MI.

## STAR★Methods

### Key resources table

**Table:** 

REAGENT or RESOURCE	SOURCE	IDENTIFIER
**Deposited data**		

Studies For Meta-analysis	PubMed and Embase	The studies included are referenced in Table 1

**Software and algorithms**		

STATA software version 13.1 (StataCorp LP)	Downloaded STATA Software	https://www.stata.com/products/
Microsoft Word 2021	Downloaded Miscrosoft Word Software	https://www.microsoft.com/en-us/microsoft-365/word

### Resource availability

#### Lead contact

Hector M. Garcia-Garcia MD, PhD.

MedStar Washington Hospital Center, 110 Irving St NW, Washington DC 2 0010 (USA), Phone: 001 (202) 877–7754, e-mail: hect2701@gmail.com.

#### Materials availability

This study is a meta-analysis and did not use or generate any reagents.

### Experimental model and study participant details

Our study does not use experimental models typical in the life sciences.

### Methods details

We performed a meta-analysis which included randomized clinical trials (RCTs) and observational studies. Inclusion criteria was the studies comparing FFR-guided PCI with non-physiology-guided revascularization strategies and availability of clinical outcome data (Data S1). We also excluded the studies comparing FFR-guided PCI with optimal medical therapy and studies using physiology assessment different from FFR. The risk of bias assessment is outlined it Tables S2 and S3. The search strategy, selection, and data extraction were performed in accordance with The Cochrane Collaboration and PRISMA guidelines. The primary outcome was all-cause mortality. Secondary outcomes were myocardial infarction (MI), repeat revascularization and major adverse cardiac events (MACE).

#### Methods

##### Search strategy and selection criteria

Randomized clinical trials (RCTs) and observational studies including patients with coronary artery disease (CAD) undergoing coronary revascularization were evaluated for inclusion in this meta-analysis. Eligible studies had to satisfy the following pre-specified inclusion criteria: 1) studies comparing FFR-guided PCI with non-physiology-guided revascularization strategies (angiography- or intracoronary imaging-guided PCI or CABG) and 2) availability of clinical outcome data. Exclusion criteria were: 1) studies comparing FFR-guided PCI with optimal medical therapy and 2) studies using physiology assessment different from FFR.

Search strategy, study selection, data extraction, and data analysis were performed in accordance with The Cochrane Collaboration and the Preferred Reporting Items for Systematic Reviews and Meta-Analyses (PRISMA) guidelines.^17^

In March 2023, we searched PubMed and Embase. In addition, we employed backward snowballing (i.e., review of references from identified articles and pertinent reviews). The search strategy is available in the supplementary appendix. This study is registered with PROSPERO (CRD42022320132).

##### Data extraction

Three investigators (JSS, JF, and JMS) independently assessed studies for possible inclusion, with the senior investigator (HGG) resolving discrepancies. Non-relevant articles were excluded based on title and abstract. The same investigators independently extracted data on study design, measurements, patient characteristics, and outcomes, using a standardized data-extraction form. Data extraction conflicts were discussed and resolved with the senior investigator (HGG).

Data about authors, year of publication, inclusion and exclusion criteria, sample size, baseline patients’ features, endpoint definitions, effect estimates, and follow-up time were collected.

##### Outcomes of interest

The pre-specified primary endpoint was all-cause death. Secondary clinical endpoints were myocardial infarction (MI), repeat revascularization, and major adverse cardiac events (MACEs). For clinical studies not reporting MACE, data on major adverse cardiac and cerebrovascular events were used. Each endpoint was assessed according to the definitions reported in the original study protocols, as summarized in Table S1.

##### Risk of bias

The risk of bias in each study has been assessed using the revised Cochrane risk of bias tool (RoB 2.0)^18^ for RCTs and the Risk of Bias in Non-randomized Studies of Interventions assessment tool from Cochrane handbook (ROBINS-I)^19^ for observational studies. Three investigators (JSS, JF, and JMS) independently assessed five domains of bias in RCTs: (1) randomization process, (2) deviations from intended interventions, (3) missing outcome data, (4) measurement of the outcome, and (5) selection of the reported results. The same investigators independently assessed seven domains of bias in observational studies: (1) confounding, (2) selection of participants, (3) classification of interventions, (4) deviations from intended interventions, (5) missing outcome data, (6) measurement of the outcome, and (7) selection of the reported results **(**Tables S2 and S3).

##### Statistical analysis

Odds ratios (ORs) and 95% confidence intervals (CIs) were calculated using the DerSimonian and Laird random-effects model, with the estimate of heterogeneity being taken from the Mantel-Haenszel method. The number of patients needed to treat (NNT) to prevent one event was calculated from weighted estimates of pooled ORs from the random-effects meta-analytic model. The presence of heterogeneity among studies was evaluated with the Cochran Q chi-squared test, with p ≤ 0.10 considered of statistical significance, and using the I^2^ test to evaluate inconsistency. A value of 0% indicates no observed heterogeneity, and larger values indicate increasing heterogeneity. I^2^ values of ≤25%, ≤50%, and >50% indicated low, moderate, and high heterogeneity, respectively. Publication bias and small study effect were assessed using funnel plots. The presence of publication bias was investigated with Harbord and Egger tests, and by visual estimation with funnel plots. Two separate pre-specified subgroup analyses according to: 1) the control revascularization strategy (i.e., angiography-guided, intracoronary imaging-guided, CABG) and 2) study design (i.e., RCT vs. observational studies) were performed. OR with 95% CI was reported in each subgroup. To assess the interaction between these potential effect modifiers and treatment, a random-effects meta-regression analysis with the “empirical Bayes” (Paule-Mandel) method to estimate the between-study variance Tau^2^ and the Hartung-Knapp-Sidik-Jonkman adjustment was performed. The meta-regression coefficient and its corresponding p value were reported (the statistical level of significance was two-tailed p < 0.1). Furthermore, we assessed the presence of interaction between number of diseased vessels, follow-up duration, and percentage of population presenting with acute coronary syndrome (ACS) and treatment for all endpoints by performing meta-regression analyses with the same method as earlier. Pre-specified sensitivity analyses were performed by iteratively removing one study at a time to confirm that our findings were not driven by any single study. We calculated 95% prediction intervals for the effect estimates of each outcome to present the expected range of true effects in a future trial, based on the extent of heterogeneity. Analyses were performed according to the intention-to-treat principle. The statistical level of significance was two-tailed p < 0.05. Statistical analyses were performed with the Stata software version 13.1 (StataCorp LP, College Station).

### Quantification and statistical analysis

DerSimonian and Laird random-effects model was used to calculate odds ratios (OR) and 95% confidence intervals (CI) and estimate of heterogeneity was taken from the Mantel-Haenszel method. The presence of heterogeneity among studies was evaluated with the Cochran Q chi-square test, and using the I^2^ test to evaluate inconsistency. We used Harbord and Egger tests to identify the presence of publication bias in addition to visual estimation with funnel plots (Figures S1–S4). A random-effects meta-regression analysis (Table S4) with the “empirical Bayes” (Paule-Mandel) method was employed to estimate the between study variance Tau^2^ and the Hartung-Knapp-Sidik-Jonkman adjustment was performed to study the interaction between potential effect modifiers and treatment. A leave-one-out sensitivity analysis was performed for the outcomes by removing one study at a time to confirm that our findings were not driven by any single study (Table S5. Leave-one sensitivity analysis for all-cause death, related to STAR Methods (Quantification and Statistical Analysis), Table S6. Leave-one sensitivity analysis for myocardial infarction, related to STAR Methods (Quantification and Statistical Analysis), Table S7. Leave-one sensitivity analysis for repeat revascularization, related to STAR Methods (Quantification and Statistical Analysis), Table S8. Leave-one sensitivity analysis for MACE, related to STAR Methods (Quantification and Statistical Analysis)). Stratified analyses according to the revascularization strategy and study design was also performed (Figures S5–S6). The statistical level of significance was two-tailed p < 0.05. Publication bias was detected for all-cause mortality as shown by Harbord test (p = 0.005). Stata software version 13.1 was used for statistical analyses.

### Additional resources

Our study has not generated or contributed to a new website and it is not part of a clinical trial.

## Data Availability

This meta-analysis used data from published studies which are referenced in the manuscript. The methods used for meta-analysis are referenced are explained in the ‘methods’ section.

## Supplemental information

Document S1. Figures S1–S6 and Data S1

Table S1. Definition of key endpoints, related to Table 1

Table S2. Risk of bias assessment for observational studies, related to STAR Methods

Table S3. Risk of bias assessment for randomized clinical trials, related to STAR Methods

Table S4. Meta-regression results, related to STAR Methods (Quantification and Statistical Analysis)

Table S5. Leave-one sensitivity analysis for all-cause death, related to STAR Methods (Quantification and Statistical Analysis)

Table S6. Leave-one sensitivity analysis for myocardial infarction, related to STAR Methods (Quantification and Statistical Analysis)

Table S7. Leave-one sensitivity analysis for repeat revascularization, related to STAR Methods (Quantification and Statistical Analysis)

Table S8. Leave-one sensitivity analysis for MACE, related to STAR Methods (Quantification and Statistical Analysis)

## References

[1] Kim J.E., Koo B.K. (2012). Fractional flow reserve: the past, present and future. Korean Circ. J..

[2] Lawton J.S., Tamis-Holland J.E., Bangalore S., Bates E.R., Beckie T.M., Bischoff J.M., Bittl J.A., Cohen M.G., DiMaio J.M., Creighton W.D., Writing Committee Members (2022). 2021 ACC/AHA/SCAI guideline for coronary artery revascularization. J. Am. Coll. Cardiol..

[3] Neumann F.-J., Sousa-Uva M., Ahlsson A., Alfonso F., Banning A.P., Benedetto U., Byrne R.A., Collet J.-P., Falk V., Head S.J. (2019). 2018 ESC/EACTS guidelines on myocardial revascularization. Eur. Heart J..

[4] Bech G.J., De Bruyne B., Pijls N.H., de Muinck E.D., Hoorntje J.C., Escaned J., Stella P.R., Boersma E., Bartunek J., Koolen J.J., Wijns W. (2001). Fractional flow reserve to determine the appropriateness of angioplasty in moderate coronary stenosis. Circulation.

[5] Tonino P.A.L., De Bruyne B., Pijls N.H.J., Siebert U., Ikeno F., van' t Veer M., Klauss V., Manoharan G., Engstrøm T., Oldroyd K.G. (2009). Fractional flow reserve versus angiography for guiding percutaneous coronary intervention. N. Engl. J. Med..

[6] De Bruyne B., Pijls N.H.J., Kalesan B., Barbato E., Tonino P.A.L., Piroth Z., Jagic N., Möbius-Winkler S., Rioufol G., Witt N. (2012). Fractional flow reserve–guided PCI versus medical therapy in stable coronary disease. N. Engl. J. Med..

[7] Jentzer J.C., Scutella M., Pike F., Fitzgibbon J., Krehel N.M., Kowalski L., Callaway C.W., Rittenberger J.C., Reynolds J.C., Barsness G.W., Dezfulian C. (2018). Early coronary angiography and percutaneous coronary intervention are associated with improved outcomes after out of hospital cardiac arrest. Resuscitation.

[8] Deharo P., Ducrocq G., Bode C., Cohen M., Cuisset T., Mehta S.R., Pollack C., Wiviott S.D., Elbez Y., Sabatine M.S., Steg P.G. (2017). Timing of angiography and outcomes in high-risk patients with non–ST-segment–elevation myocardial infarction managed invasively. Circulation.

[9] Mentias A., Sarrazin M.V., Saad M., Panaich S., Kapadia S., Horwitz P.A., Girotra S. (2020). Long-term outcomes of coronary stenting with and without use of intravascular ultrasound. JACC Cardiovasc. Interv..

[10] Kim Y., Bae S., Johnson T.W., Son N.H., Sim D.S., Hong Y.J., Kim S.W., Cho D.K., Kim J.S., Kim B.K. (2022). Role of intravascular ultrasound-guided percutaneous coronary intervention in optimizing outcomes in acute myocardial infarction. J. Am. Heart Assoc..

[11] Jones D.A., Rathod K.S., Koganti S., Hamshere S., Astroulakis Z., Lim P., Sirker A., O’Mahony C., Jain A.K., Knight C.J. (2018). Angiography alone versus angiography plus optical coherence tomography to guide percutaneous coronary intervention: outcomes from the Pan-London PCI cohort. JACC Cardiovasc. Interv..

[12] Meneveau N., Souteyrand G., Motreff P., Caussin C., Amabile N., Ohlmann P., Morel O., Lefrançois Y., Descotes-Genon V., Silvain J. (2016). Optical coherence tomography to optimize results of percutaneous coronary intervention in patients with non–ST-elevation acute coronary syndrome. Circulation.

[13] Volleberg R., Mol J.-Q., van der Heijden D., Meuwissen M., van Leeuwen M., Escaned J., Holm N., Adriaenssens T., van Geuns R.J., Tu S. (2021). Optical coherence tomography and coronary revascularization: from indication to procedural optimization. Trends Cardiovasc. Med..

[14] Zhang D., Lv S., Song X., Yuan F., Xu F., Zhang M., Yan S., Cao X. (2015). Fractional flow reserve versus angiography for guiding percutaneous coronary intervention: a meta-analysis. Heart.

[15] Puymirat E., Cayla G., Simon T., Steg P.G., Montalescot G., Durand-Zaleski I., le Bras A., Gallet R., Khalife K., Morelle J.-F. (2021). Multivessel PCI guided by FFR or angiography for myocardial infarction. N. Engl. J. Med..

[16] Rioufol G., Dérimay F., Roubille F., Perret T., Motreff P., Angoulvant D., Cottin Y., Meunier L., Cetran L., Cayla G. (2021). Fractional flow reserve to guide treatment of patients with multivessel coronary artery disease. J. Am. Coll. Cardiol..

[17] Liberati A., Altman D.G., Tetzlaff J., Mulrow C., Gøtzsche P.C., Ioannidis J.P.A., Clarke M., Devereaux P.J., Kleijnen J., Moher D. (2009). The PRISMA statement for reporting systematic reviews and meta-analyses of studies that evaluate healthcare interventions: explanation and elaboration. BMJ.

[18] Sterne J.A.C., Savović J., Page M.J., Elbers R.G., Blencowe N.S., Boutron I., Cates C.J., Cheng H.-Y., Corbett M.S., Eldridge S.M. (2019). RoB 2: a revised tool for assessing risk of bias in randomised trials. BMJ.

[19] Sterne J.A., Hernán M.A., Reeves B.C., Savović J., Berkman N.D., Viswanathan M., Henry D., Altman D.G., Ansari M.T., Boutron I. (2016). ROBINS-I: a tool for assessing risk of bias in non-randomised studies of interventions. BMJ.

[20] Layland J., Oldroyd K.G., Curzen N., Sood A., Balachandran K., Das R., Junejo S., Ahmed N., Lee M.M.Y., Shaukat A. (2015). Fractional flow reserve vs. angiography in guiding management to optimize outcomes in non-ST-segment elevation myocardial infarction: the British Heart Foundation FAMOUS-NSTEMI randomized trial. Eur. Heart J..

[21] Koo B.-K., Hu X., Kang J., Zhang J., Jiang J., Hahn J.-Y., Nam C.-W., Doh J.-H., Lee B.-K., Kim W. (2022). Fractional flow reserve or intravascular ultrasonography to guide PCI. N. Engl. J. Med..

[22] Fröhlich G.M., Redwood S., Rakhit R., MacCarthy P.A., Lim P., Crake T., White S.K., Knight C.J., Kustosz C., Knapp G. (2014). Long-term survival in patients undergoing percutaneous interventions with or without intracoronary pressure wire guidance or intracoronary ultrasonographic imaging: a large cohort study. JAMA Intern. Med..

[23] Hu P., Tang M.Y., Song W.C., Jiang J., Sun Y., Liu X.B., Li C.L., Hu X.Y., Wang J.A. (2015). Fractional flow reserve guided percutaneous coronary intervention improves clinical outcome with reduced cost in contemporary clinical practice. Chin. Med. J..

[24] Li J., Elrashidi M.Y., Flammer A.J., Lennon R.J., Bell M.R., Holmes D.R., Bresnahan J.F., Rihal C.S., Lerman L.O., Lerman A. (2013). Long-term outcomes of fractional flow reserve-guided vs. angiography-guided percutaneous coronary intervention in contemporary practice. Eur. Heart J..

[25] Lunardi M., Scarsini R., Venturi G., Pesarini G., Pighi M., Gratta A., Gottin L., Barbierato M., Caprioglio F., Piccoli A. (2019). Physiological versus angiographic guidance for myocardial revascularization in patients undergoing transcatheter aortic valve implantation. J. Am. Heart Assoc..

[26] Parikh R.V., Liu G., Plomondon M.E., Sehested T.S.G., Hlatky M.A., Waldo S.W., Fearon W.F. (2020). Utilization and outcomes of measuring fractional flow reserve in patients with stable ischemic heart disease. J. Am. Coll. Cardiol..

[27] Puymirat E., Peace A., Mangiacapra F., Conte M., Ntarladimas Y., Bartunek J., Vanderheyden M., Wijns W., De Bruyne B., Barbato E. (2012). Long-term clinical outcome after fractional flow reserve-guided percutaneous coronary revascularization in patients with small-vessel disease. Circ. Cardiovasc. Interv..

[28] Di Gioia G., De Bruyne B., Pellicano M., Bartunek J., Colaiori I., Fiordelisi A., Canciello G., Xaplanteris P., Fournier S., Katbeh A. (2020). Fractional flow reserve in patients with reduced ejection fraction. Eur. Heart J..

[29] Fearon W.F., Zimmermann F.M., De Bruyne B., Piroth Z., van Straten A.H.M., Szekely L., Davidavičius G., Kalinauskas G., Mansour S., Kharbanda R. (2022). Fractional flow reserve–guided PCI as compared with coronary bypass surgery. N. Engl. J. Med..

[30] Levine G.N., Bates E.R., Blankenship J.C., Bailey S.R., Bittl J.A., Cercek B., Chambers C.E., Ellis S.G., Guyton R.A., Hollenberg S.M. (2011). 2011 ACCF/AHA/SCAI guideline for percutaneous coronary intervention: a report of the American College of Cardiology Foundation/American Heart Association Task Force on practice guidelines and the society for cardiovascular angiography and interventions. Circulation.

[31] van Nunen L.X., Zimmermann F.M., Tonino P.A.L., Barbato E., Baumbach A., Engstrøm T., Klauss V., MacCarthy P.A., Manoharan G., Oldroyd K.G. (2015). Fractional flow reserve versus angiography for guidance of PCI in patients with multivessel coronary artery disease (FAME): 5-year follow-up of a randomised controlled trial. Lancet.

[32] Xaplanteris P., Fournier S., Pijls N.H.J., Fearon W.F., Barbato E., Tonino P.A.L., Engstrøm T., Kääb S., Dambrink J.-H., Rioufol G. (2018). Five-year outcomes with PCI guided by fractional flow reserve. N. Engl. J. Med..

[33] Elbadawi A., Dang A.T., Hamed M., Eid M., Prakash Hiriyur Prakash M., Saleh M., Gad M., Mamas M.A., Rahman F., Elgendy I.Y. (2022). FFR- versus angiography-guided revascularization for nonculprit stenosis in stemi and multivessel disease: a network meta-analysis. JACC Cardiovasc. Interv..

[34] Elbadawi A., Sedhom R., Dang A.T., Gad M.M., Rahman F., Brilakis E.S., Elgendy I.Y., Jneid H. (2022). Fractional flow reserve versus angiography alone in guiding myocardial revascularisation: a systematic review and meta-analysis of randomised trials. Heart.

[35] Burzotta F., Leone A.M., Aurigemma C., Zambrano A., Zimbardo G., Arioti M., Vergallo R., De Maria G.L., Cerracchio E., Romagnoli E. (2020). Fractional flow reserve or optical coherence tomography to guide management of angiographically intermediate coronary stenosis: a single-center trial. JACC Cardiovasc. Interv..

[36] Hu M.J., Tan J.S., Yin L., Zhao Y.Y., Gao X.J., Yang J.G., Yang Y.J. (2022). Clinical outcomes following hemodynamic parameter or intravascular imaging-guided percutaneous coronary intervention in the era of drug-eluting stents: an updated systematic review and bayesian network meta-analysis of 28 randomized trials and 11,860 patients. Front. Cardiovasc. Med..

[37] Di Gioia G., Soto Flores N., Franco D., Colaiori I., Sonck J., Gigante C., Kodeboina M., Bartunek J., Vanderheyden M., Van Praet F. (2020). Coronary artery bypass grafting or fractional flow reserve-guided percutaneous coronary intervention in diabetic patients with multivessel disease. Circ. Cardiovasc. Interv..

[38] Maron D.J., Hochman J.S., Reynolds H.R., Bangalore S., O’Brien S.M., Boden W.E., Chaitman B.R., Senior R., López-Sendón J., Alexander K.P. (2020). Initial invasive or conservative strategy for stable coronary disease. N. Engl. J. Med..

[39] O’Fee K., Deych E., Ciani O., Brown D.L. (2021). Assessment of nonfatal myocardial infarction as a surrogate for all-cause and cardiovascular mortality in treatment or prevention of coronary artery disease: a meta-analysis of randomized clinical trials. JAMA Intern. Med..

[40] Del Rosario M., Guduguntla V., Wang T.Y. (2021). Nonfatal myocardial infarction-poor surrogate for mortality. JAMA Intern. Med..

[41] Sanz-Sánchez J., McFadden E., Garcia-Garcia H.M. (2022). The importance of using the appropriate model for systematic reviews and meta-analyses. JAMA Intern. Med..

[42] Smits P.C., Abdel-Wahab M., Neumann F.J., Boxma-de Klerk B.M., Lunde K., Schotborgh C.E., Piroth Z., Horak D., Wlodarczak A., Ong P.J. (2017). Fractional flow reserve-guided multivessel angioplasty in myocardial infarction. N. Engl. J. Med..

[43] Engstrøm T., Kelbæk H., Helqvist S., Høfsten D.E., Kløvgaard L., Holmvang L., Jørgensen E., Pedersen F., Saunamäki K., Clemmensen P. (2015). Complete revascularisation versus treatment of the culprit lesion only in patients with ST-segment elevation myocardial infarction and multivessel disease (DANAMI-3—PRIMULTI): an open-label, randomised controlled trial. Lancet.

[44] Hakeem A., Edupuganti M.M., Almomani A., Pothineni N.V., Payne J., Abualsuod A.M., Bhatti S., Ahmed Z., Uretsky B.F. (2016). Long-term prognosis of deferred acute coronary syndrome lesions based on nonischemic fractional flow reserve. J. Am. Coll. Cardiol..

[45] Cerrato E., Mejía-Rentería H., Dehbi H.-M., Ahn J.-M., Cook C., Dupouy P., Baptista S.B., Raposo L., Van Belle E., Götberg M. (2020). Revascularization deferral of nonculprit stenoses on the basis of fractional flow reserve. JACC Cardiovasc. Interv..

[46] Escaned J., Ryan N., Mejía-Rentería H., Cook C.M., Dehbi H.-M., Alegria-Barrero E., Alghamdi A., Al-Lamee R., Altman J., Ambrosia A. (2018). Safety of the deferral of coronary revascularization on the basis of instantaneous wave-free ratio and fractional flow reserve measurements in stable coronary artery disease and acute coronary syndromes. JACC Cardiovasc. Interv..

[47] Liou K.P., Ooi S.-Y.M., Hoole S.P., West N.E.J. (2019). Fractional flow reserve in acute coronary syndrome: a meta-analysis and systematic review. Open Heart.

[48] Claessen B.E., van Wijk D.F. (2020). FFR in the setting of ACS: can we do better?. JACC Cardiovasc. Interv..

[49] Sanz-Sánchez J., Stefanini G.G. (2022). Timing and completeness of revascularisation in acute coronary syndromes. Heart.

[50] Kedhi E., Berta B., Roleder T., Hermanides R.S., Fabris E., IJsselmuiden A.J.J., Kauer F., Alfonso F., von Birgelen C., Escaned J. (2021). Thin-cap fibroatheroma predicts clinical events in diabetic patients with normal fractional flow reserve: the COMBINE OCT–FFR trial. Eur. Heart J..

[51] de la Torre Hernandez J.M., Lopez-Palop R., Garcia Camarero T., Carrillo Saez P., Martin Gorria G., Frutos Garcia A., Arnaez Corada B., Cordero Fort A., Gomez Delgado J.M., Agudo Quilez P. (2013). Clinical outcomes after intravascular ultrasound and fractional flow reserve assessment of intermediate coronary lesions. Propensity score matching of large cohorts from two institutions with a differential approach. EuroIntervention..

[52] Park S.H., Jeon K.H., Lee J.M., Nam C.W., Doh J.H., Lee B.K., Rha S.W., Yoo K.D., Jung K.T., Cho Y.S. (2015). Long-Term Clinical Outcomes of Fractional Flow Reserve-Guided Versus Routine Drug-Eluting Stent Implantation in Patients With Intermediate Coronary Stenosis: Five-Year Clinical Outcomes of DEFER-DES Trial. Circ. Cardiovasc. Interv..

[53] Chen S.L., Ye F., Zhang J.J., Xu T., Tian N.L., Liu Z.Z., Lin S., Shan S.J., Ge Z., You W. (2015). Randomized comparison of FFR-guided and angiography-guided provisional stenting of true coronary bifurcation lesions: the DKCRUSH-VI trial (Double Kissing Crush Versus Provisional Stenting Technique for Treatment of Coronary Bifurcation Lesions VI). JACC Cardiovasc. Interv..

[54] Elkady A.O., Abdelghany M., Diab R., Ezz A., Elagha A.A. (2021). Total versus staged versus functional revascularization in NSTEACS patients with multivessel disease. Egypt. Heart J..

[55] Di Gioia G., Pellicano M., Toth G.G., Casselman F., Adjedj J., Van Praet F., Ferrara A., Stockman B., Degrieck I., Bartunek J. (2016). Fractional flow reserve-guided revascularization in patients with aortic stenosis. Am. J. Cardiol..

[56] Koo B.K., Park K.W., Kang H.J., Cho Y.S., Chung W.Y., Youn T.J., Chae I.H., Choi D.J., Tahk S.J., Oh B.H. (2008). Physiological evaluation of the provisional side-branch intervention strategy for bifurcation lesions using fractional flow reserve. Eur. Heart J..

[57] Nam C.W., Yoon H.J., Cho Y.K., Park H.S., Kim H., Hur S.H., Kim Y.N., Chung I.S., Koo B.K., Tahk S.J. (2010). Outcomes of percutaneous coronary intervention in intermediate coronary artery disease: fractional flow reserve-guided versus intravascular ultrasound-guided. JACC Cardiovasc. Interv..

[58] Quintella E.F., Ferreira E., Azevedo V.M.P., Araujo D.V., Sant'Anna F.M., Amorim B., Albuquerque D.C.d. (2019). Clinical outcomes and cost-effectiveness analysis of FFR compared with angiography in multivessel disease patient. Arq. Bras. Cardiol..

[59] Di Serafino L., De Bruyne B., Mangiacapra F., Bartunek J., Agostoni P., Vanderheyden M., Scognamiglio G., Heyndrickx G.R., Wijns W., Barbato E. (2013). Long-term clinical outcome after fractional flow reserve- versus angio-guided percutaneous coronary intervention in patients with intermediate stenosis of coronary artery bypass grafts. Am. Heart J..

[60] Wongpraparut N., Yalamanchili V., Pasnoori V., Satran A., Chandra M., Masden R., Leesar M.A. (2005). Thirty-month outcome after fractional flow reserve-guided versus conventional multivessel percutaneous coronary intervention. Am. J. Cardiol..

[61] Zhang Z., Li K., Tian J. (2016). Efficacy and safety outcomes of fractional flow reserve in guiding clinical therapy of non-ST-segment elevation myocardial infarction compared with angiography alone in elderly Chinese patients. Clin. Interv. Aging.

